# Whole Transcriptome Sequencing Analyses Reveal Molecular Markers of Blood Pressure Response to Thiazide Diuretics

**DOI:** 10.1038/s41598-017-16343-z

**Published:** 2017-11-22

**Authors:** Ana Caroline C. Sá, Amy Webb, Yan Gong, Caitrin W. McDonough, Somnath Datta, Taimour Y. Langaee, Stephen T. Turner, Amber L. Beitelshees, Arlene B. Chapman, Eric Boerwinkle, John G. Gums, Steven E. Scherer, Rhonda M. Cooper-DeHoff, Wolfgang Sadee, Julie A. Johnson

**Affiliations:** 1 Center for Pharmacogenomics and Department of Pharmacotherapy and Translational Research, University of Florida, Gainesville, FL USA; 2 Graduate Program in Genetics and Genomics, University of Florida, Gainesville, FL USA; 3 Department of Biomedical Informatics, College of Medicine, The Ohio State University, Columbus, OH USA; 4 Department of Biostatistics, University of Florida, Gainesville, FL USA; 5 Division of Nephrology and Hypertension, Mayo Clinic, Rochester, MN USA; 6 Division of Endocrinology, Diabetes and Nutrition, University of Maryland, Baltimore, MD USA; 7 Department of Medicine, University of Chicago, Chicago, IL USA; 8 Division of Epidemiology, University of Texas at Houston, Houston, TX USA; 9 Department of Community Health and Family Medicine, University of Florida College of Medicine, Gainesville, FL USA; 10 Human Genome Sequencing Center, Baylor College of Medicine, Houston, TX USA; 11 Division of Cardiovascular Medicine, Department of Medicine, University of Florida College of Medicine, Gainesville, FL USA; 12 Center for Pharmacogenomics, Department of Cancer Biology and Genetic, College of Medicine, Ohio State University, Columbus, OH USA

## Abstract

Thiazide diuretics (TD) are commonly prescribed anti-hypertensives worldwide. However, <40% of patients treated with thiazide monotherapy achieve BP control. This study uses whole transcriptome sequencing to identify novel molecular markers associated with BP response to TD. We assessed global RNA expression levels in whole blood samples from 150 participants, representing patients in the upper and lower quartile of BP response to TD from the Pharmacogenomic Evaluation of Antihypertensive Responses (PEAR) (50 whites) and from PEAR-2 (50 whites and 50 blacks). In each study cohort, we performed poly-*A* RNA-sequencing in baseline samples from 25 responders and 25 non-responders to hydrochlorothiazide (HCTZ) or chlorthalidone. At FDR adjusted p-value < 0.05, 29 genes were differentially expressed in relation to HCTZ or chlorthalidone BP response in whites. For each differentially expressed gene, replication was attempted in the alternate white group and PEAR-2 blacks. *CEBPD* (meta-analysis p = 1.8 × 10^−11^) and *TSC22D3* (p = 1.9 × 10^−9^) were differentially expressed in all 3 cohorts, and explain, in aggregate, 21.9% of response variability to TD. This is the first report of the use of transcriptome-wide sequencing data to identify molecular markers of antihypertensive drug response. These findings support *CEBPD* and *TSC22D3* as potential biomarkers of BP response to TD.

## Introduction

Hypertension (HTN) affects approximately 80 million adults in the United States and one billion worldwide^1,2^. HTN is the most important modifiable risk factor for cardiovascular and renal diseases, and the use of antihypertensive medications is associated with decreased morbidity and mortality^3^. Despite the availability of numerous blood pressure (BP) lowering medications from different drug classes with different mechanisms of action, only about half of patients treated with antihypertensive medications achieve appropriate BP control^4,5^.

Thiazide diuretics are among the most commonly prescribed antihypertensive medications in the US, with more than 50 million hydrochlorothiazide (HCTZ) prescriptions in 2014^6^, and likely double that when combination products are considered. Thiazides are a first-line option for HTN treatment, yet patients’ responses vary widely and less than 40% of patients achieve BP control^4,7^. This reveals that the inter-individual variability in BP response to TD is likely to contribute to the suboptimal BP control.

In the past 10 years, pharmacogenomic studies have increased our understanding of the potential role of specific genetic variants with BP response to antihypertensive drugs^8–10^. Recently, two replicated regions, one in PRKCA (protein kinase C, alpha) and the other one near GNAS (G protein alpha subunit), were identified with potentially clinically relevant effects on BP response to HCTZ^11^. Despite success with the GWAS approach, stringent cutoffs for statistical significance (P < 5.0 × 10^−8^) relative to the sample sizes available in hypertension pharmacogenomics cohorts limit the detection of additional polymorphisms influencing BP response to antihypertensive drugs. In addition, these results suggest the involvement of multiple genes, each contributing only a fraction to the overall genetic influence on hypertension.

The study of RNA transcriptomes by deep sequencing^12^ (RNA-Seq) represents an alternative approach to identifying candidate genes. RNA transcripts are the most proximate phenotype that reflects the integration of multiple genetic variants in *cis* and in *trans*, in addition to restraints imposed by gene networks and pathways^13,14^. As a result, RNA levels can serve as proximate indicators of a disease state or drug response, with greater sensitivity than genetic variants by themselves. RNA-Seq has brought relevant qualitative and quantitative improvements to transcriptome analysis, offering an unprecedented level of resolution and a unique tool to simultaneously investigate different layers of transcriptome complexity. RNA levels and allelic-specific RNA expression, the latter a sensitive indicator of *cis-*regulatory variants^15^, can serve to discover regulatory genetic variants associated with expression and RNA processing, thereby adding to our understanding of factors that influence phenotype. Thus, in this study, we aim to identify genes/transcripts associated with BP response to thiazide diuretics and investigate allele specific expression within these genes, as a mechanism to potentially explain the detected differences in gene expression.

## Results

In order to study inter-individual variability in expression that potentially impacts BP response to TD, we generated transcriptome sequencing data from 150 hypertensive participants treated with HCTZ or chlorthalidone, and data passed quality control procedures on 149. For each sample, RNA-Seq reads were mapped to the human genome, resulting in 11–63 million mapped reads per sample. Of those, 79–95% of the reads were uniquely mapped. These and other mapping statistics are presented in the Supplementary Table 1.

Table 1 displays baseline and demographic characteristics from PEAR and PEAR-2 participants selected for RNA-Sequencing. Age, gender and baseline BP among responders and non-responders to HCTZ were similar. However, these characteristics did differ significantly between PEAR-2 white and black responders and non-responders to chlorthalidone, as shown in Table 1.

**Table 1:** Characteristics of PEAR and PEAR-2 participants classified as responder and non-responders for RNA-Seq differential expression and allele specific expression analyses.

**Characteristics**	Whites (n = 99)				Blacks (n = 50)	
	HCTZ		Chlorthalidone		Chlorthalidone	
	**Responders (n = 24)**	**Non-responders (n = 25)**	**Responders (n = 25)**	**Non-responders (n = 25)**	**Responders (n = 25)**	**Non-responders (n = 25)**
**Age**	48 ± 12	48 ± 8	53 ± 8	48 ± 10	52 ± 8	50 ± 10
**Female, n (%)**	11 (44%)	10 (40%)	15 (75%)*	5 (25%)*	12 (48%)	12 (48%)
**Baseline DBP**	93 ± 5	94 ± 4	97 ± 6*	93 ± 5*	98 ± 6*	93 ± 4*
**Baseline SBP**	146 ± 10	144 ± 10	152 ± 11*	144 ± 9*	152 ± 10*	146 ± 10*
**DBP response to TD**	−9 ± 6***	0.06 ± 4***	−14 ± 4***	−0.2 ± 2***	−17 ± 4***	−1.4 ± 3***
**SBP response to TD**	−12 ± 6***	−0.9 ± 6***	−22 ± 7***	−1.5 ± 5***	−27 ± 7***	−4.4 ± 5***

Mean and Standard Deviation values for the continuous variables were presented. SBP: systolic blood pressure; DBP: diastolic blood pressure; TD: thiazide diuretics. ***P < 0.001.

Mean changes of serum potassium concentrations and uric acid levels in non-responders were determined before and after treatment with HCTZ and chlorthalidone (Table 2), with the premise that if the cause of the nonresponse in BP lowering was nonadherence, then it would be unlikely that there would be any observed adverse metabolic responses typically seen with TD treatment^16–18^. Change in serum potassium and uric acid, from baseline to after treatment, was assessed with paired t-tests. After treatment with HCTZ and chlorthalidone, there were significant reductions in serum potassium and significant increases in serum uric acid in participants classified as non-responders (Table 2), consistent with previously reported adverse metabolic effects of TD^19,20^, suggesting non-adherence with TDs in the group of BP non-responders is unlikely.

**Table 2:** Potassium and uric acid mean changes in participants classified as non-responders after treatment with HCTZ and chlorthalidone.

Parameters	Whites				Blacks	
	Non-responders to HCTZ (n = 25)		Non-responders to Chlorthalidone (n = 25)		Non-responders to Chlorthalidone (n = 25)	
	Mean change ± s.d.	P value	Mean change ± s.d.	P value	Mean change ± s.d.	P value
**Serum K** ^**+**^ **(mEq/L)**	−0.2 ± 0.4	0.016	−0.6 ± 0.4	2.0E-07	−0.45 ± 0.6	0.001
**Serum uric acid, mg/dl**	0.9 ± 1.0	9.6E-05	1.1 ± 1.0	2.8E-05	1.1 ± 1.4	5.6E-04

P values represent the comparison between baseline and the end of the monotherapy.

### Differential mRNA Expression

**Figure 1:**
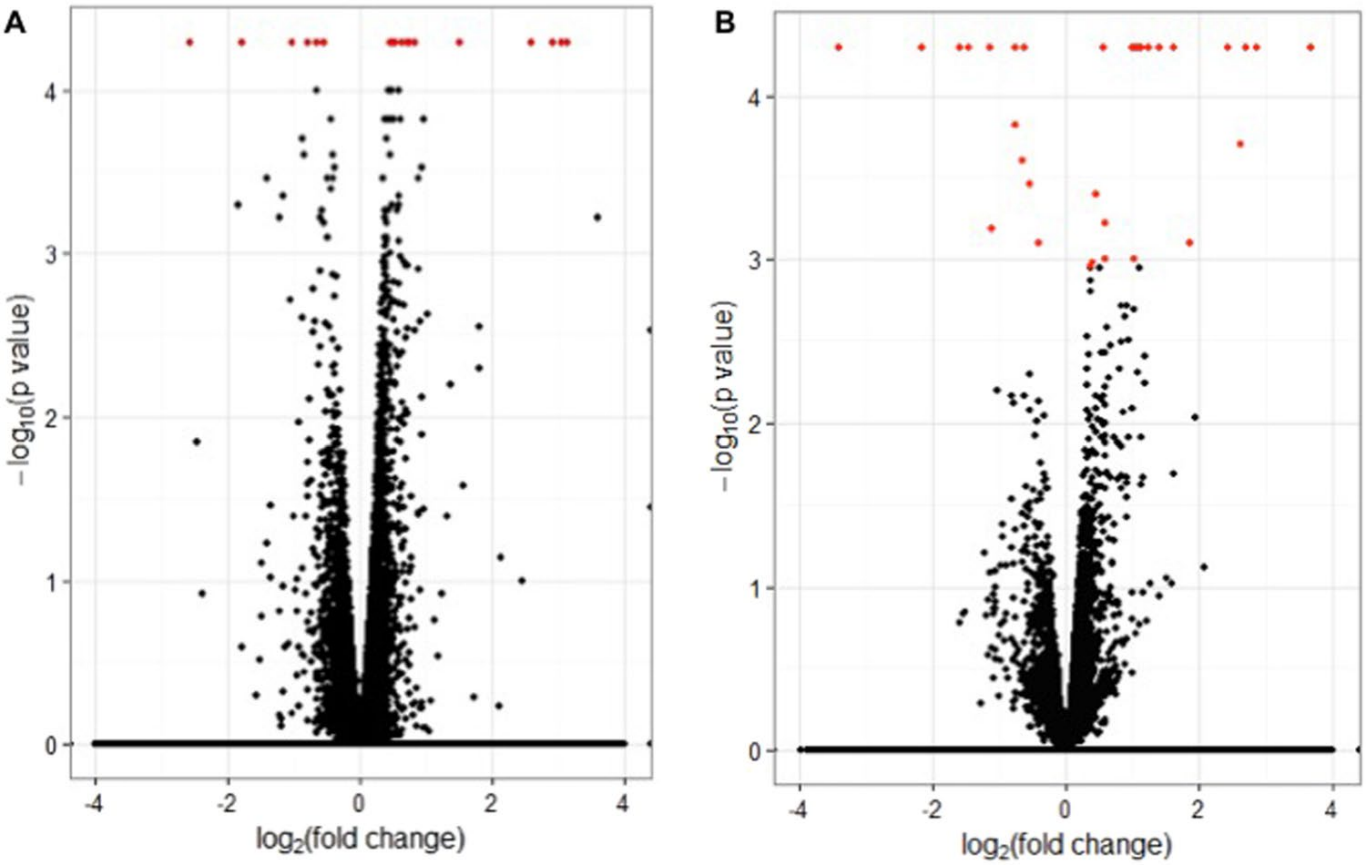
Volcano plots comparing gene expression between responders and non-responders to HCTZ in PEAR whites (**A**) and chlorthalidone in PEAR-2 whites (**B**). Plot of log-fold changes versus log-p-values of probability of differential expression. Each gene is represented on the plot as a single dot. The red dots represent genes that passed the statistical threshold of FDR adjusted p-value < 0.05.

We identified genes differentially expressed between responders and non-responders to HCTZ and chlorthalidone, in PEAR and PEAR-2 whites. Overall, 12,948 and 13,160 transcripts were detected with FPKM ≥ 1 in the responders or non-responders to HCTZ and chlorthalidone, respectively. At Q value < 0.05, 11 and 18 unique genes were differentially expressed in PEAR and PEAR-2 whites, respectively (Fig. 1 and Supplementary Tables 2 and 3).

### Validation of gene expression associations with BP response to TD

**Figure 2:**
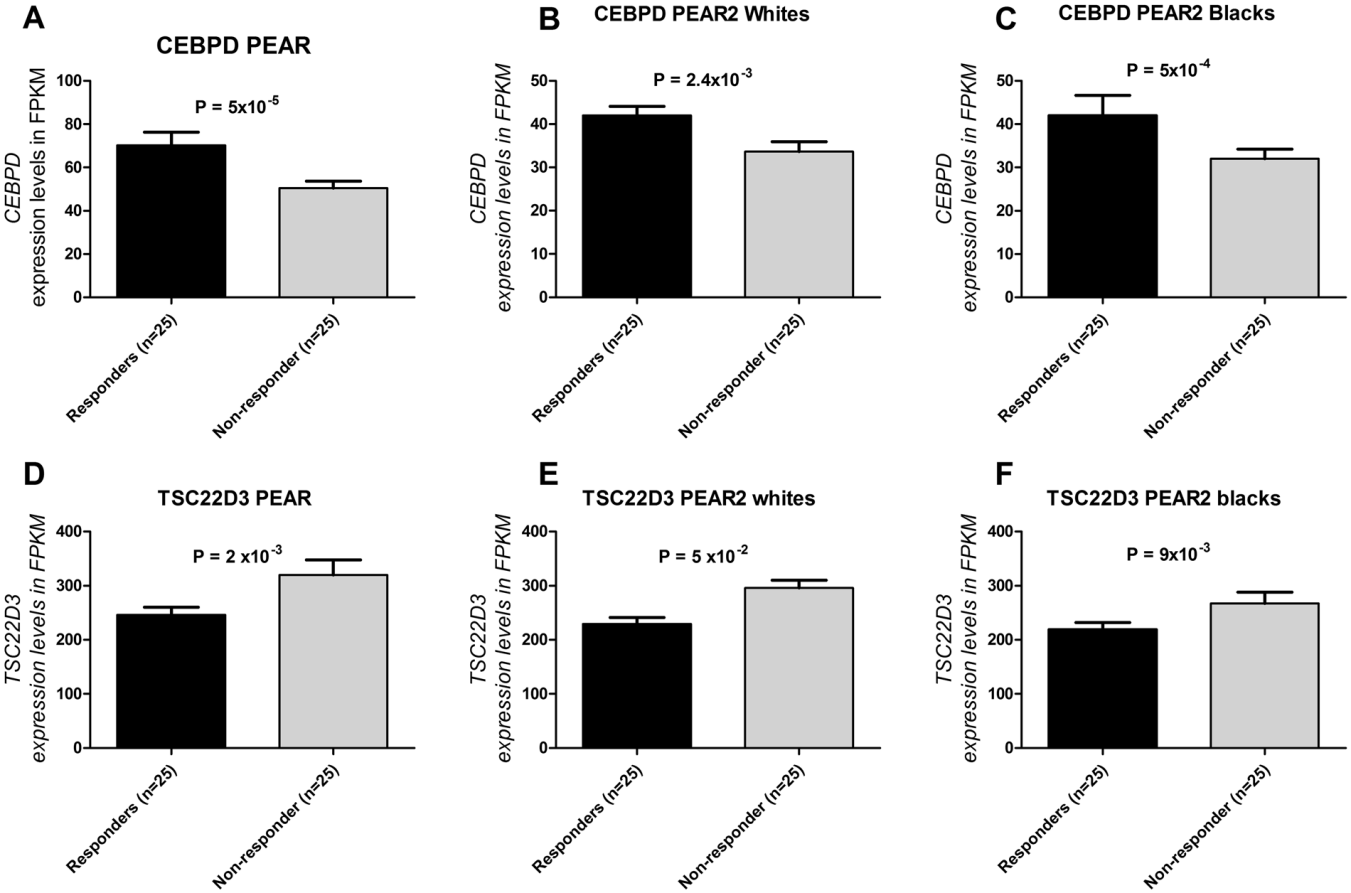
Plots showing CEBPD and TSC22D3 baseline expression levels between thiazide responders compared to non-responders in the PEAR and PEAR-2 RNA-Seq analyses. (**A**) CEBPD in PEAR (whites). (**B**) CEBPD in PEAR-2 whites. (**C**) CEBPD in PEAR-2 blacks. (**D**) TSC22D3 in PEAR. (**E**) TSC22D3 in PEAR-2 whites. (**F)** TSC22D3 in PEAR-2 blacks. Abundance comparisons between thiazide diuretics responders and non-responders were carried using Cufflinks v2.2.1. Error bars indicate standard error of the mean. HCTZ: hydrochlorothiazide, FPKM: fragments per kilobase per million reads.

In order to validate the differential expression results, replication in the other white group and in PEAR-2 blacks for each gene differentially expressed in PEAR or PEAR-2 whites was attempted (Supplementary Tables 2 and 3). *CEBPD* and *TSC22D3* showed statistically significant differences in expression in the same direction (FPKM in responders compared to non-responders) in all 3 groups tested (Table 3). The results from the meta-analysis displayed in the Table 2 revealed that association of *CEBPD* and *TSC22D3* expression with BP response to TD achieved transcriptome-wide significance (*CEBPD*: P = 1.8 × 10^−11^ and *TSC22D3*: P = 1.9 × 10^−9^). Higher *CEBPD* expression was observed in responders than non-responders to TD across blacks and whites and the two different drugs in the TD drug class: HCTZ and chlorthalidone (Fig. 2A–C). In contrast, *TSC22D3* showed increased expression levels in non-responders to TD consistently in PEAR whites and PEAR-2 white and black participants (Fig. 2D–F). These results identify *CEBPD* and *TSC22D3* transcripts robustly associated with BP response to TD.

**Table 3:** Genes differentially expressed between responders and non-responders to HCTZ and chlorthalidone in all 3 cohorts, with consistent direction and transcriptome-wide statistical significance when meta-analyzed.

Genes	HCTZ whites		Chlorthalidone whites		Chlorthalidone blacks		Meta-analysis
	Fold Change	P-Value	Fold Change	P-value	Fold Change	P-value	P-value
**CEBPD**	1.4	**5.0E-05**	1.2	**2.4E-03**	1.3	**5.3E-04**	**1.8E-11**
**TSC22D3**	0.8	**1.8E-03**	0.8	**4.87E-02**	0.8	**8.8E-03**	**1.9E-09**

Fold change corresponds to gene expression levels in responders divided by levels in non-responders, in fragments per kilobase per million reads (FPKM).

The differential expression results with edgeR, including age, gender and baseline BP in the statistical model, revealed similar effect sizes, fold change in expression between responders and non-responders, when compared to the results with Cuffdiff for *CEBPD* (Supplementary Table 4), although the p value of this association was not as low. The edgeR analyses for *TSC22D3* were not statistically significant (Supplementary Table 4).

Since *TSC22D3* is located in the X chromosome, we also investigated the overall expression levels (FPKM) of this gene in PEAR and PEAR-2 male and female participants (Supplementary Figure 1). There were no sex-specific differences detected in *TSC22D3* expression (PEAR: P = 0.09, PEAR-2 whites: P = 0.37 and PEAR-2 blacks: P = 0.39), which suggests that X inactivation escape was not the cause of the observed *TSC22D3* differential expression results.

### Biomarker evaluation with model building and validation

**Figure 3:**
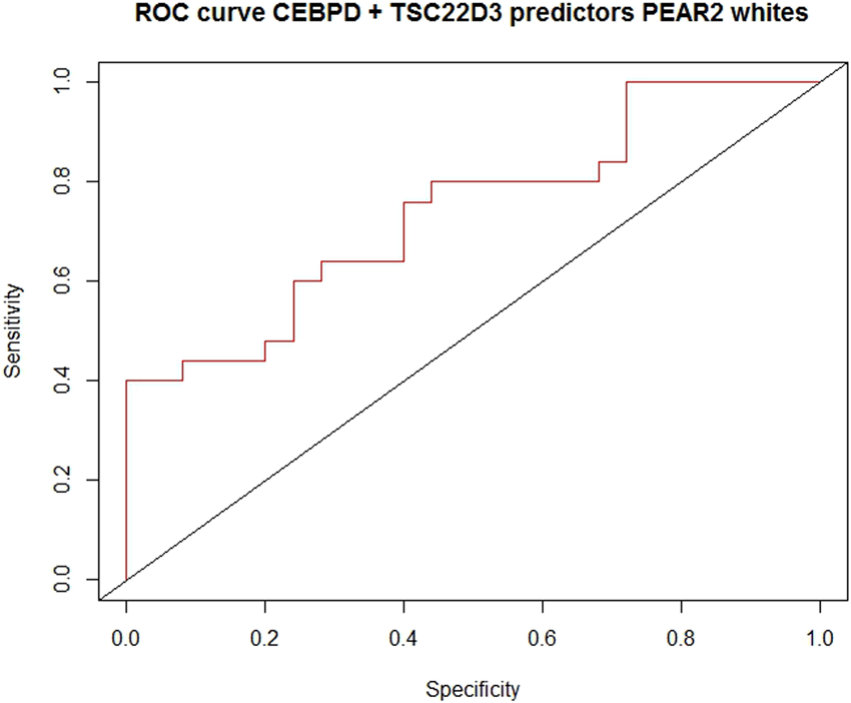
Receiver operator curve for assessment of logistic regression model prediction in PEAR-2 whites. Model includes *TSC22D3* and *CEBPD* expression measures in Fragments per Kilobase of Exon per Million mapped (FPKM).

Multiple logistic regression analysis revealed that *TSC22D3* or *CEBPD* gene expression alone were not statistically significant predictors of BP response to TD using PEAR whites as the derivation cohort. However, the combination of these genes in the model was statistically significant (P = 0.01), and explained 21.9% of the variability in drug response to TD in the derivation cohort. For independent assessment of this model in PEAR-2 whites, the area under the curve was 0.74, indicating a good prediction model for BP response to TD (Fig. 3).

### Allele Specific Expression Analysis

We also sought to determine whether there was evidence of *cis*-acting regulation for *CEBPD* and *TSC22D3*. However, we were not able to achieve sufficient number of heterozygous (>2) or enough RNA-Seq coverage (>30 reads) for ASE analysis in these candidate gene regions.

## Discussion

To the best of our knowledge, this is the first study to investigate the association of global gene expression levels with BP response to antihypertensive drugs. Unlike other studies profiling gene expression, here, RNA-Seq data from whole blood samples obtained from 3 cohorts of participants selected based on the extremes of BP response to TD were included: PEAR whites treated with HCTZ and PEAR-2 whites and blacks treated with chlorthalidone. The application of robust methods to quantify gene expression, with high sequencing resolution and available data for the replication and validation of the results reveal the potential to provide previously unrecognized insights into BP regulation and responses to antihypertensive drugs.

Herein, 29 genes were differentially expressed (Q value < 0.05) between white participants classified as responders and non-responders to HCTZ or chlorthalidone. Among them, *CEBPD* and *TSC22D3* were differentially expressed between responders and non-responders in three different cohorts treated with thiazide diuretics, with consistent directional fold change in whites treated with HCTZ and whites and blacks treated with chlorthalidone.

The top differentially expressed gene, *CEBPD*, (meta-analysis P-value = 1.8 × 10^−11^), is located at chromosome 8p11.2-p11.1 and encodes the transcription factor CCAAT/enhancer binding protein delta. Previously, the expression of *CEBPD* was associated with strain-specific differential transcription activation of Platelet-Derived Growth Factor-α Receptor (PDGF-αR) expression between spontaneously hypertensive (SHR) and normotensive (Wistar-Kyoto) rats^21^. This strong bimodal (all versus none) strain-specific effect in *PDGF-αR* expression suggests that PDGF-αR and its transcription-regulating factors are significantly related to genetic hypertension through proliferation and migration of vascular smooth muscle cells^21^. Additionally, members of the CEBP family of transcription factors, especially CEBPB (beta) and CEBPD, showed regulatory effects on the expression of the *angiotensinogen (AGT)* gene by increasing the promoter activity mediated by interleukin 6^22^. *CEBPD* is known to facilitate the binding of other transcription factors and contribute to chromatin remodeling not only for the genes mentioned here^23^, with documented impact in hypertension, but also genes involved in immune and inflammatory responses^24^. Therefore, further experiments will be valuable to understand the regulatory mechanisms by which CEBPD is involved in BP response to TD.

Differences in *TSC22D3* expression was also strongly associated with BP response to HCTZ and chlorthalidone (meta-analysis P-value = 1.9 × 10^−9^). *TSC22D3*, located at the chromosome Xq22.3, encodes the anti-inflammatory protein glucocorticoid (GC)-induced leucine zipper, also known as *Gilz*. *TSC22D3* expression is stimulated by glucocorticoids^25^, interleukin 10^26^ and aldosterone^27^, and the latter plays a role in sodium homeostasis in the distal nephron via activation of the apical epithelial sodium channel (EnaC)^28^. Aldosterone dose-dependent activation of *TSC22D3* mediates the inhibition of the negative feedback mechanism, regulating the EnaC deactivation, which ultimately drives sodium retention^27^. Further experimental validation will be crucial to close the link between TSC22D3 and BP regulation with TD.

Although in humans the majority of X-linked genes are subject to X-inactivation, at least 15% of them are thought to escape X-inactivation, being expressed from both the active and inactive X chromosomes in women^29^. Due to the localization of *TSC22D3* in the X chromosome, the association between gene expression levels with gender (Supplementary Figure 1) was tested. There was no statistically significant difference in expression levels between genders. Collectively, these results suggest that an effect of X inactivation escape can be dismissed.

There are some limitations worthy of mention. First, our sample size for RNA-Seq differential expression and ASE analysis may have restricted the power to identify additional signals, as well as to validate some of the findings; however, the power of the number of samples tested was enhanced by taking an extreme phenotype approach. Second, using whole blood samples for RNA-Seq data analysis may have also limited the detection of some tissue-specific genes/regulatory mechanisms. However, it is challenging to select only one tissue in order to investigate gene expression as a marker of BP regulation since drug response to anti-HTN might arise from a variety of target tissues such as vasculature, heart, brain, or kidney. Not only are these tissues difficult to access in relatively healthy patients, as hypertensive patients are, but it is not obvious which tissue should be used. Thus whole blood is a surrogate for multiple tissues, recognizing the limitations of tissue specific expression with this approach.

In conclusion, this is the first report of whole transcriptome sequencing analysis to identify genes potentially involved in the phenotype of antihypertensive drug response. More specifically, differences in *CEBPD* and *TSC22D3* expression associated with BP response to HCTZ and chlorthalidone in 3 unique cohorts were identified. Additional experiments are needed to demonstrate the mechanisms by which*, CEBPD* and *TSC22D3* may influence BP response to TD.

## Methods

### Study Participants

The primary analysis of this study included clinical data and whole blood samples from hypertensive participants from the Pharmacogenomic Evaluation of Antihypertensive Responses (PEAR) and PEAR-2 studies (NCT00246519, NCT01203852 www.clinicaltrials.gov). Details of these studies were previously published^30^. In brief, PEAR was a multicenter, randomized clinical trial with one of the primary aims to evaluate the role of genetics on BP response of HCTZ and/or atenolol treated patients. PEAR recruited 768 study participants with uncomplicated HTN from the University of Florida (Gainesville, FL), Emory University (Atlanta, GA), and the Mayo Clinic (Rochester, MN). These participants were randomized to receive monotherapy of either the thiazide diuretic HCTZ, or the beta-blocker atenolol for a period of 9 weeks. Fasting blood (including DNA and RNA) and urine samples were collected at baseline (untreated), after 9 weeks of monotherapy, and after 9 weeks of combination therapy (HCTZ + atenolol). BP response measurements were assessed using office, home, and 24-hour ambulatory BP and then a composite BP response was constructed^31^.

PEAR-2 was a prospective, multi-center, sequential monotherapy clinical trial, which recruited a hypertensive population with similar characteristics to the one in PEAR. One of its primary aims was to investigate the role of genetics on metoprolol, a beta-blocker, and chlorthalidone, a thiazide-like diuretic. Details of this prospective, clinical trial were previously published^32^. Briefly, 417 hypertensive participants had at least a 4-weeks washout period prior to each active treatment period with metoprolol (beta-blocker) and then chlorthalidone (thiazide diuretic). Home and clinic BP measurements, adverse metabolic effects, RNA and DNA from whole blood, and urine samples were collected.

All study participants from PEAR and PEAR-2 provided written informed consent. The Institutional Review Boards at the University of Florida, Emory University, and the Mayo Clinic approved both PEAR and PEAR-2 studies, which were conducted in accordance with the principles of the Declaration of Helsinki and the US Code of the Federal Regulations for Protection of Human Subjects.

### Gene expression profile with RNA-Seq

RNA-Seq was performed in 150 PEAR whites and PEAR-2 white and black participants, selected based on the differences in their BP response to HCTZ and chlorthalidone treatment, respectively. Sample selection was based on BP responses to either HCTZ or chlorthalidone in the top and bottom quartiles from each of the three cohorts and participants were classified as poor BP responders (non-responders) and good BP responders (responders).

Total RNA was from whole blood samples using the PAXgene Blood RNA kit IVD (Qiagen, Valenica, CA), then mRNA was selected using poly(A) selection protocol with Sera-Mag Magnetic Oligo(dT) Beads (Illumina, San Diego,CA) and fragmented to a mean length ~ 120 to 180 base pairs. Strand-specific complementary DNA libraries were prepared and sequenced on an Illumina HiSeq. 2000, performed at Baylor Human Genome Sequencing Center in Texas. One of the samples from HCTZ responders did not achieve enough yield of libraries for adequate performance in sequencing.

The paired-end 100 bp reads generated were uniquely mapped to the human reference genome (hg19) using TopHat v2.0.10^33^ allowing for four reads mismatches, read edit distance of six, one mismatch in the anchor region of a spliced read, and a maximum of five multi-hits. PCR duplicates were removed using Picard (http://picard.sourceforge.net) MarkDuplicates option. Transcript structure assembly was performed using Cufflinks v2.2.1 on each sample. Gene expression levels (in Fragments per Kilobase of Exon per Million mapped, FPKM) were calculated by considering per-isoform FPKM measurements carried out with Cuffdiff v2.2.1. Expression levels < 1 FPKM fall below the threshold for mRNA abundance required for protein detection, and therefore were not included in this analysis^34–36^.

Additionally, alternative tools were applied for differential expression analysis with the purpose to include age, gender and baseline diastolic BP in the statistical model for association with BP response to TD. With BAM files from TopHat 2 alignments, the htseq-count function from the HTSeq bioconductor package^37^ was applied to directly count the number of reads for assigned to the known human genes (Gencode gene annotation release 18). Then, these read counts were modeled to a Negative Binomial distribution using a generalized linear model in edgeR^38^. Recent independent comparison studies for differential expression analysis have shown that no single method is likely to perform favorably for all datasets^39–41^. In our study, we followed the expert recommendation^42^ to perform differential expression analyses with more than one method: using Cufflinks/Cuffdiff and HTSeq/edgeR.

### Statistical Methods

The primary data analysis for this study was performed in whites treated with HCTZ or chlorthalidone. Whole transcriptome expression levels were quantified by measuring read counts that overlap protein coding genes (count matrix) and Fragments per Kilobase of transcript per Million mapped reads (FPKM). A t-test was applied in order to assess the statistical significance for the observed differences in expression levels between responders and non-responders to TD. False discovery rate (FDR) adjusted p-values (Q value) < 0.05 were considered statistically significant.

In order to validate the association of gene expression differences with BP response to TD, we aimed to replicate the finding in PEAR-2 blacks and the alternate group of whites for each gene differentially expressed in PEAR and PEAR-2 whites. The *a priori* criteria for validation was Q value < 0.05 (considering the subset of genes differentially expressed) and consistent fold change direction (up or down regulation of expression) in all three groups: 1) whites treated with HCTZ, and 2) whites and 3) blacks treated with chlorthalidone.

The differential expression results from each study cohort were combined in a meta-analysis, using standardized p-values to follow the assumption of the Fisher p-value combination method implemented by the R package MetaRNASeq.^43^. We considered that genes with meta-analysis p-values < 2.0 × 10^−6^ (0.05/25,000) achieved transcriptome-wide association with BP response to TD.

### Biomarker evaluation with model building and validation

To evaluate whether *TSC22D3* and *CEBPD* robustly predict BP response to TD, PEAR participants were assigned into the derivation cohort for logistic regression model building. PEAR-2 whites constituted the validation cohort, in which area under the receiver operator curve was calculated in the R ROCR package^44^ for model evaluation. *TSC22D3* and *CEBPD* expression measures in FPKM were used for this analysis.

### Allele Specific Expression (ASE) Analysis

We also tested for allelic mRNA expression imbalance in the upstream/downstream within 2 kb of the coding region for the genes that passed the validation criteria in the differential expression analysis. The ASE analyses were conducted with heterozygous white participants from PEAR and PEAR-2 (n = 100) as our sample size in blacks (n = 50) was too small for a meaningful analysis. A personalized genome was built by substituting the reference allele with the variant allele SNP in hg19 using GATK FastaAlternateReference tool (www.software.broadinstitute.org/gatk/gatkdocs/org_broadinstitute_gatk_tools_fasta_FastaAlternateReferenceMaker.php) in order to overcome potential bias in read alignment, where reference allele reads can be preferentially aligning over alternative allele reads^15^. RNA-Seq reads were mapped using STAR v2.5.2b and a two-pass strategy. We followed the Broad Institute best practices workflow for SNP and indel calling from RNA-Seq data (https://www.broadinstitute.org/gatk/guide/article?id = 3891). For each SNP, ASE ratios were obtained from the division of reference allele counts over alternative allele reads counts. A binomial statistical test was applied to determine whether this ratio deviates from the expected 50:50, when the two alleles are expressed equally.

## Electronic supplementary material

Supplementary Information
