# Supplementary material for: Whole Transcriptome Sequencing Analyses Reveal Molecular Markers of Blood Pressure Response to Thiazide Diuretics
Source: Sci Rep. 2017 Nov 22;7:16068. doi: 10.1038/s41598-017-16343-z (PMC5700078; doi:10.1038/s41598-017-16343-z)
Supplement: Supplementary file 1 — Supplementary Information [file 41598_2017_16343_MOESM1_ESM.doc]

**SUPPLEMENTAL MATERIAL**

**Whole Transcriptome Sequencing Analyses Reveal Molecular Markers of Blood Pressure Response to Thiazide Diuretics**

Ana Caroline C. Sá^1,2^, Amy Webb^3^, Yan Gong^1^, Caitrin W. McDonough^1^, Somnath Datta^4^, Taimour Y. Langaee^1^, Stephen T. Turner^5^, Amber L. Beitelshees^6^, Arlene B. Chapman^7^, Eric Boerwinkle^8^, John G. Gums^9^, Steven E. Scherer^10^, Rhonda M. Cooper-DeHoff^1,11^, Wolfgang Sadee^12^, Julie A. Johnson^1, 2,11^

1Center for Pharmacogenomics and Department of Pharmacotherapy and Translational Research, University of Florida, Gainesville, FL, USA

2Graduate Program in Genetics and Genomics, University of Florida, Gainesville, FL, USA

3Department of Biomedical Informatics, College of Medicine, The Ohio State University, Columbus, OH, USA

4Department of Biostatistics, University of Florida, Gainesville, FL, USA

5Division of Nephrology and Hypertension, Mayo Clinic, Rochester, MN, USA

6Division of Endocrinology, Diabetes and Nutrition, University of Maryland, Baltimore, MD, USA

7 Department of Medicine, University of Chicago, Chicago, IL, USA

8Division of Epidemiology, University of Texas at Houston, Houston, TX, USA

9Department of Community Health and Family Medicine, University of Florida College of Medicine, Gainesville, FL, USA

10Human Genome Sequencing Center, Baylor College of Medicine, Houston, TX, USA.

11Division of Cardiovascular Medicine, Department of Medicine, University of Florida College of Medicine, Gainesville, FL, USA

12Center for Pharmacogenomics, Department of Cancer Biology and Genetic, College of Medicine, Ohio State University, Columbus, OH, USA

Corresponding author:

Julie A. Johnson, Pharm.D.

Center for Pharmacogenomics, Department of Pharmacotherapy and Translational Research, College of Pharmacy, University of Florida

P.O.Box 100484 Gainesville, FL 32610-0486

352-273-6309(phone)/352-273-6306 (fax)

Email: johnson@cop.ufl.edu

**Table S1**. Summary of mapping statistics from PEAR and PEAR-2 RNA-Seq alignment with Tophat2

| **Mapping characteristics** | **PEAR whites mean(range)** | **PEAR-2 whites mean(range)** | **PEAR-2 blacks mean(range)** |
| --- | --- | --- | --- |
| Reads aligned | 27,032,558  (14,247,017-44,816,469) | 33,709,514  (15,437,743-52,173,426) | 33,737,127  (11,287,549-63,147,881) |
| Uniquely mapped (%) | 93.0  (88.4-94.9) | 93.5  (91.3-95.1) | 92.1  (78.6-95.2) |
| Remaining after duplicate removal (%) | 47.3  (15.0-63.9) | 61.3  (29.0-80.5) | 58.7  (28.1-84.9) |
| Known junctions (%) | 86.8  (82.9-90.5) | 85.1  (79.9-89.9) | 84.8%  (76.7-91.5) |
| Reads aligned to exonic regions (%) | 65.5  (57.5-70.8) | 61.6  (55.7-68.5) | 60.5  (42.7-70.2) |
|  |  |  |  |

**Table S2**. Genes differentially expressed in PEAR whites treated with HCTZ at Q-value < 0.05 and gene expression results in PEAR-2 whites or blacks treated with chlorthalidone

|  | **PEAR HCTZ whites** | | | **PEAR-2 Chlorthalidone whites** | | | **PEAR-2 Chlorthalidone blacks** | | |
| --- | --- | --- | --- | --- | --- | --- | --- | --- | --- |
| **GENE** | **FOLD CHANGE** | ***P*** | ***Q*** | **FOLD CHANGE** | ***P*** | ***Q*** | **FOLD CHANGE** | ***P*** | ***Q*** |
| TSEN34 | 1.5 | 5.0E-05 | 0.034 | 1.07 | 2.0E-01 | 0.259 | 1.35 | 3.0E-04 | 0.002 |
| CEBPD | 1.4 | 5.0E-05 | 0.034 | 1.25 | 2.4E-03 | 0.031 | 1.31 | 5.3E-04 | 0.002 |
| TIGD3 | 1.4 | 5.0E-05 | 0.034 | 1.19 | 4.8E-02 | 0.171 | 1.37 | 1.8E-03 | 0.006 |
| VNN1 | 1.7 | 5.0E-05 | 0.034 | 1.15 | 9.6E-02 | 0.208 | 1.29 | 1.3E-02 | 0.033 |
| TSPO | 1.4 | 5.0E-05 | 0.034 | 1.05 | 2.8E-01 | 0.327 | 1.20 | 2.0E-02 | 0.044 |
| CDC42EP2 | 1.4 | 5.0E-05 | 0.034 | 0.98 | 4.2E-01 | 0.416 | 1.19 | 3.3E-02 | 0.062 |
| RHOB | 1.4 | 5.0E-05 | 0.034 | 1.12 | 8.2E-02 | 0.208 | 1.12 | 8.0E-02 | 0.130 |
| TRGC1 | 0.6 | 5.0E-05 | 0.034 | 1.12 | 1.6E-01 | 0.238 | 1.09 | 2.2E-01 | 0.292 |
| FCRL6 | 0.7 | 5.0E-05 | 0.034 | 1.21 | 5.3E-02 | 0.171 | 1.08 | 2.6E-01 | 0.306 |
| CHI3L1 | 1.6 | 5.0E-05 | 0.034 | 0.91 | 1.6E-01 | 0.238 | 0.95 | 3.2E-01 | 0.351 |
| IGHG1 | 0.6 | 5.0E-05 | 0.034 | 1.15 | 1.1E-01 | 0.213 | 1.00 | 5.0E-01 | 0.496 |

Fold change corresponds to gene expression levels in responders divided by levels in non-responders, in fragments per kilobase per million reads (FPKM). Highlighted genes that passed specified criteria for validation: consistent gene expression fold change and statistical significance (Q < 0.05).

*One sided p-value based on a one-sided hypothesis tested in the validation cohorts

**Table S3**. Genes differentially expressed in PEAR-2 whites treated with chlorthalidone at Q-value < 0.05 and gene expression results in PEAR whites or PEAR-2 blacks

|  | **PEAR-2 WHITES** | | | **PEAR WHITES** | | | **PEAR-2 BLACKS** | | |
| --- | --- | --- | --- | --- | --- | --- | --- | --- | --- |
| **GENE** | **FOLD CHANGE** | ***P*** | ***Q*** | **FOLD CHANGE** | ***P*** | ***Q*** | **FOLD CHANGE** | ***P*** | ***Q*** |
| TRIT1 | 1.71 | 5.0E-05 | 0.004 | 1.09 | 2.3E-01 | 0.494 | 0.37 | 2.5E-05 | 0.0001 |
| SERINC5 | 0.05 | 5.0E-05 | 0.004 | 1.10 | 3.7E-01 | 0.494 | 0.11 | 2.5E-05 | 0.0001 |
| TFCP2 | 0.37 | 5.0E-05 | 0.004 | 1.02 | 4.2E-01 | 0.494 | 0.15 | 2.5E-05 | 0.0001 |
| PPP2R5C | 0.50 | 5.0E-05 | 0.004 | 1.02 | 4.6E-01 | 0.494 | 0.33 | 2.5E-05 | 0.0001 |
| METTL23 | 2.75 | 5.0E-05 | 0.004 | 1.01 | 4.8E-01 | 0.494 | 2.79 | 2.5E-05 | 0.0001 |
| METTL6 | 10.71 | 5.0E-05 | 0.004 | 1.00 | 4.9E-01 | 0.494 | 0.45 | 2.5E-05 | 0.0001 |
| TSC22D3 | 0.78 | 1.1E-03 | 0.049 | 0.77 | 1.8E-03 | 0.018 | 0.82 | 8.8E-03 | 0.0221 |
| LTF | 1.55 | 5.0E-05 | 0.004 | 0.88 | 1.2E-01 | 0.470 | 1.42 | 1.1E-02 | 0.0251 |
| GPR56 | 0.76 | 1.1E-03 | 0.047 | 1.33 | 3.8E-03 | 0.025 | 0.87 | 5.3E-02 | 0.1067 |
| IGHA2 | 1.47 | 3.5E-04 | 0.020 | 1.12 | 1.2E-01 | 0.470 | 0.88 | 9.5E-02 | 0.1458 |
| BPI | 1.70 | 1.5E-04 | 0.011 | 1.04 | 3.8E-01 | 0.494 | 1.25 | 8.1E-02 | 0.1458 |
| PHACTR4 | 1.57 | 2.5E-04 | 0.016 | 1.00 | 4.9E-01 | 0.494 | 1.18 | 9.4E-02 | 0.1458 |
| FGFBP2, PROM1 | 0.73 | 4.0E-04 | 0.023 | 1.33 | 1.8E-04 | 0.004 | 0.92 | 1.6E-01 | 0.2320 |
| AP3S2 | 1.58 | 2.5E-04 | 0.016 | 0.99 | 4.9E-01 | 0.494 | 0.91 | 2.3E-01 | 0.3005 |
| OCIAD2 | 0.08 | 5.0E-05 | 0.004 | 1.02 | 4.6E-01 | 0.494 | 0.89 | 2.4E-01 | 0.3008 |
| LRBA | 0.48 | 5.0E-05 | 0.004 | 1.12 | 2.6E-01 | 0.494 | 0.94 | 3.8E-01 | 0.4413 |
| SLC37A3 | 0.37 | 5.0E-05 | 0.004 | 0.90 | 2.2E-01 | 0.494 | 1.03 | 4.2E-01 | 0.4655 |
| PHKB | 3.05 | 5.0E-05 | 0.004 | 1.02 | 4.6E-01 | 0.494 | 0.99 | 4.7E-01 | 0.4743 |

Fold change corresponds to gene expression levels in responders divided by levels in non-responders, in fragments per kilobase per million reads (FPKM). Highlighted genes that passed specified criteria for validation: consistent gene expression fold change and statistical significance (Q > 0.05).

*One sided p-value based on a one-sided hypothesis tested in the validation cohorts

**Table S4**. Differences in baseline expression levels for CEBPD and TSC22D3 between thiazide diuretics responders and non-responders in PEAR and PEAR-2 with adjustment for age, gender and baseline blood pressure

|  | **HCTZ Whites** | | **Chlorthalidone Whites** | | **Chlorthalidone Blacks** | |
| --- | --- | --- | --- | --- | --- | --- |
| **Genes** | **Fold Change** | ***P* value** | **Fold Change** | ***P* value*** | **Fold Change** | ***P* value*** |
| CEBPD | 1.45 | 0.0337 | 1.25 | 0.02 | 1.21 | 0.05 |
| SERINC5 | 1.00 | 0.9772 | 0.94 | 0.17 | 0.98 | 0.43 |
| TSC22D3 | 1.32 | 0.1248 | 1.14 | 0.06 | 1.12 | 0.10 |

Generalized linear model implemented in edgeR^21^

Fold change corresponds to gene expression levels in responders divided by levels in non-responders, in fragments per kilobase per million reads (FPKM).

*One sided p-value based on a one-sided hypothesis tested in the validation cohorts


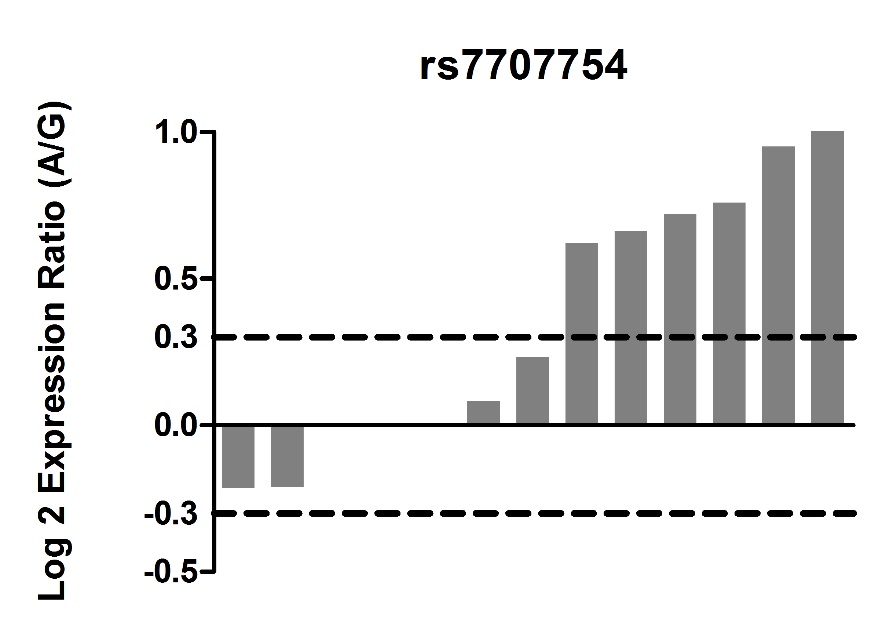


**Supplementary Figure 1**. Allele-specific expression ratios (major allele over minor allele) in *SERINC5* rs7707754. Each bar represents the magnitude and direction of allelic expression imbalance (AEI) for one heterozygous individual indicated on a log2 scale. The horizontal dashed lines at log2 expression 0.3 and -0.3 represent the pre-established threshold for AEI.


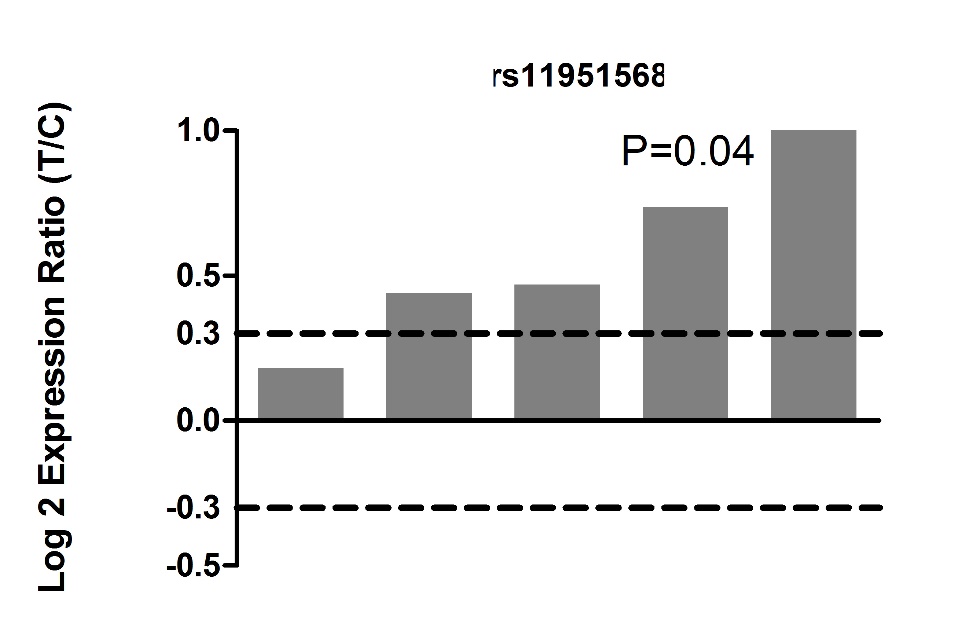


**Supplementary Figure 2**. Allele-specific expression ratios (major allele over minor allele) in *SERINC5* rs11951568. Each bar represents the magnitude and direction of allelic expression imbalance (AEI) for one heterozygous individual indicated on a log2 scale. The horizontal dashed lines at log2 expression 0.3 and -0.3 represent the pre-established threshold for AEI.
